# Diminished social motivation in early psychosis is associated with polygenic liability for low vitamin D

**DOI:** 10.1038/s41398-024-02750-0

**Published:** 2024-01-18

**Authors:** Alex Hatzimanolis, Sarah Tosato, Mirella Ruggeri, Doriana Cristofalo, Leonidas Mantonakis, Lida-Alkisti Xenaki, Stefanos Dimitrakopoulos, Mirjana Selakovic, Stefania Foteli, Ioannis Kosteletos, Ilias Vlachos, Rigas-Filippos Soldatos, Nikos Nianiakas, Irene Ralli, Konstantinos Kollias, Angeliki-Aikaterini Ntigrintaki, Pentagiotissa Stefanatou, Robin M. Murray, Evangelos Vassos, Nikos C. Stefanis

**Affiliations:** 1 Department of Psychiatry, Medical School, National and Kapodistrian University of Athens, Eginition University Hospital, Athens, Greece; 2 Neurobiology Research Institute, Theodore-Theohari Cozzika Foundation, Athens, Greece; 3 Section of Psychiatry, Department of Neuroscience, Biomedicine and Movement Sciences, University of Verona, Verona, Italy; 4 Department of Psychosis Studies, Institute of Psychiatry, Psychology and Neuroscience, King’s College London, London, UK; 5 National Institute for Health Research, Mental Health Biomedical Research Centre at South London and Maudsley NHS Foundation Trust and King’s College, London, UK; 6 Department of Social, Genetic and Developmental Psychiatry Centre, Institute of Psychiatry, Psychology and Neuroscience, King’s College London, London, UK

**Keywords:** Schizophrenia, Genomics

## Abstract

Insufficiency of vitamin D levels often occur in individuals with schizophrenia and first-episode psychosis (FEP). However, it is unknown whether this represents a biological predisposition, or it is essentially driven by illness-related alterations in lifestyle habits. Lower vitamin D has also been associated with adverse neurodevelopmental outcomes and predominant negative psychotic symptoms. This study aimed to investigate the contribution of polygenic risk score for circulating 25-hydroxyvitamin D concentration (PRS-vitD) to symptom presentation among individuals with FEP enrolled in the Athens First-Episode Psychosis Research Study (AthensFEP *n* = 205) and the Psychosis Incident Cohort Outcome Study (PICOS *n* = 123). The severity of psychopathology was evaluated using the Positive and Negative Syndrome Scale at baseline and follow-up assessments (AthensFEP: 4-weeks follow-up, PICOS: 1-year follow-up). Premorbid intelligence and adjustment domains were also examined as proxy measures of neurodevelopmental deviations. An inverse association between PRS-vitD and severity of negative symptoms, in particular lack of social motivation, was detected in the AthensFEP at baseline (*adjusted R*^2^ = 0.04, *p* < 0.001) and follow-up (*adjusted R*^2^ = 0.03, *p* < 0.01). The above observation was independently validated in PICOS at follow-up (*adjusted R*^2^ = 0.06, *p* < 0.01). No evidence emerged for a relationship between PRS-vitD and premorbid measures of intelligence and adjustment, likely not supporting an impact of lower PRS-vitD on developmental trajectories related to psychotic illness. These findings suggest that polygenic vulnerability to reduced vitamin D impairs motivation and social interaction in individuals with FEP, thereby interventions that encourage outdoor activities and social engagement in this patient group might attenuate enduring negative symptoms.

## Introduction

It is well established that 25-hydroxyvitamin D, the main circulating form of vitamin D, constitutes a neuro-steroid compound and vital nutrient involved in the regulation of multiple physiological processes, including fetal brain development [1, 2], and adult brain functioning [3]. The beneficial neuromodulatory influences of normal vitamin D levels have been widely documented, indicating that vitamin D could exert neuroprotective and neurotrophic actions [2, 4]. Further, the anti-oxidant and anti-inflammatory properties of vitamin D support its neuroprotective role in neuronal cell function [4]. Findings from animal studies have primarily implicated insufficient vitamin D levels to abnormal dopamine neuron development and differentiation [5, 6], even though multiple neurotransmitter systems could also be negatively affected in neonatal brain [7]. Reduced vitamin D levels have been widely documented in major psychiatric disorders, including broadly defined psychotic disorders [8], schizophrenia [9], bipolar disorder [10, 11], and major depressive disorder [12]. Moreover, vitamin D deficiency or insufficiency is prevalent in individuals with first-episode of psychosis (FEP) [13–16], indicating that diminished levels of vitamin D already exist at the early stages of psychotic illness.

The exact biological mechanism underlying the lack of vitamin D during early psychosis is still largely unknown. Several observational studies have demonstrated that sub-optimal vitamin D is associated with more pronounced negative symptoms, cognitive impairments, and poor functional outcomes in individuals with either FEP or chronic psychosis-spectrum disorders, in particular schizophrenia [17–22]. Similarly, individuals with FEP characterized by lower vitamin D levels exhibit less improvement of negative symptoms following treatment with antipsychotic medication [23]. Fewer studies have reported associations between vitamin D insufficiency and positive symptoms [13, 18]. Among individuals with schizophrenia, low vitamin D concentration has been associated with the occurrence of depressive symptoms, elevated suicide risk, and increased antidepressant consumption [22, 24]. Over the past years, it is a matter of debate whether vitamin D deficiency often observed in patients with psychotic disorders could be explained by as of yet unrecognizable neurobiological alterations, or it is rather attributed to social preferences, individual lifestyle habits, less sun exposure, and poor general nutrition [25]. In fact, a probable hypothesis implies that lower vitamin D levels in patients with schizophrenia could result from the experience of early life environmental adversities [18], while prenatal or neonatal vitamin D deficiency has been suggested as a potential environmental risk factor for the development of schizophrenia later in life, independently of polygenic liability for schizophrenia [9, 26].

From a genetic perspective, genome-wide correlation analyses within healthy individuals have revealed overlapping genetic underpinnings between vitamin D levels and brain-related phenotypes, including regional brain cortical thickness, intelligence, autism, major depression, bipolar disorder, and schizophrenia [27–29]. Further, independent analyses of the UK Biobank population-based dataset did not report significant relationships between genetically predicted vitamin D inadequacy and elevated risk for major psychiatric disorders or cognitive dysfunction [28, 30]. Hence, the above observations do not support a causal contribution of genetic loci associated with reduced vitamin D in the emergence of major psychiatric disorders. Conversely, it has been suggested that genetic risk for intelligence and psychotic disorders might be causally related to decreased vitamin D concentration [30]. Likewise, similar results were observed for behavioural traits linked to the aforementioned phenotypes (i.e. time spent using a computer, duration of walks, less outdoor activity), which could possibly mediate the existence of vitamin D shortage due to limited sun exposure [30].

It is stressed that until recently evidence for a genetic relationship between vitamin D insufficiency and psychotic symptom severity is lacking. To fill this gap, the present study aimed to investigate the association of polygenic risk score for vitamin D concentration with symptom presentation in two European cohorts of individuals with FEP. We hypothesized that genetic risk variants associated with lower vitamin D would predispose to more severe psychopathology. Additionally, in an attempt to further explore the potential influence of polygenic risk for reduced vitamin D on aberrant neurodevelopment, we examined premorbid intelligence and adjustment domains as proxy measures that most likely mirror impaired developmental trajectories preceding psychotic illness onset [31–33].

## Methods

### Participants

#### The Athens First-Episode Psychosis Research Study (AthensFEP)

A detailed description of the study design and clinical assessments has been provided in earlier reports [34, 35]. Briefly, as part of the AthensFEP study, a total of 225 individuals experiencing their first-episode of psychosis (FEP) were recruited from five psychiatric clinical settings throughout the metropolitan area of Athens, Greece. Inclusion criteria were the presence of FEP at age 16–45 years and exposure to antipsychotic medication for less than two weeks. Individuals with psychotic disorders due to another medical condition or acute intoxication, IQ < 70, developmental disorders, and kinship with an enrolled participant were excluded from the study. All cases were screened using the diagnostic interview for psychosis (DIP), a standardized semi-structured interview generating diagnoses according to different diagnostic algorithms, on the basis of the Operational Criteria Checklist for Psychotic Illness (OPCRIT) [36]. Clinician-based interviews were conducted at admission to the study (baseline) and following 4-weeks of treatment with antipsychotics by trained psychiatrists or neuropsychologists. In expert consensus meetings, involving the principal investigators and the research associate assigned with each case, clinical diagnoses were established at 4-weeks follow-up visit according to the International Classification of Diseases 10th Revision (ICD-10) criteria. The Research Ethics Committees of the five participating hospitals approved the study protocol. A signed informed consent was obtained before the inclusion to the study protocol.

#### The Psychosis Incident Cohort Outcome Study (PICOS)

Detailed information on study design and recruitment of patients can be found in previous reports [37, 38]. Briefly, as part of the PICOS study, a total of 123 individuals experiencing their first-episode of psychosis (FEP) were recruited from Community Mental Health Centers (CMHCs) located in the Veneto Region (North-Eastern Italy) and assessed at baseline, and after 1 and 2 years. Individuals, aged 18–54 years, were recruited if they contacted local CMHCs for the first time in their life with at least one of the following symptoms: hallucinations, delusions, qualitative speech disorder, qualitative psychomotor disorder, bizarre or grossly inappropriate behavior; or two of the following symptoms: loss of interest, initiative, and drive; social withdrawal; episodic severe excitement; purposeless destructiveness; overwhelming fear; or marked self-neglect. Individuals were excluded if they were under antipsychotic medication for more than three months, affected by a mental disorder due to a chronic medical condition, have received a diagnosis of non-psychotic illness or diagnosed with intellectual disability. Diagnosis was confirmed after six months from the inclusion to the study using the Item Group Checklist of the Schedules for Clinical Assessment in Neuropsychiatry (SCAN) interview [39]. All individuals provided written informed consent following a complete description of the study, which has been approved by the ethics committees of the coordinating center and the local participating sites.

### Clinical assessments

#### Symptom evaluation

The Positive and Negative Syndrome Scale (PANSS) [40] was administered to all participants by trained psychiatrists at baseline and after 1-month (AthensFEP) or after 1-year (PICOS) in order to assess the severity of psychopathology symptoms. PANSS derived subscale (i.e. positive, negative, general psychopathology) and total scores were examined in subsequent analyses. In addition, as current conceptualization of negative symptoms points towards a rather multidimensional construct instead of a single severity domain [41], negative symptoms were stratified in two separate sub-domains, namely expressive deficits (ED) and social amotivation (SA) [42]. Previous studies have shown that ED and SA sub-domains capture distinct dimensions of negative symptomatology [42, 43].

#### Premorbid adjustment

The Cannon-Spoor Premorbid Adjustment Scale (PAS) [44] was administered to assess premorbid adjustment (PA) in the AthensFEP study. The PAS retrospectively examines aspects of premorbid functioning across four developmental stages: childhood (up to 11 years), early adolescence (12–15 years), late adolescence (16–18 years), and adulthood (19 years and beyond); and across two domains: academic (scholastic performance, adaptation to school) and social (sociability/withdrawal, peer relationships, and socio-sexual functioning). Detailed information with regard to early life functioning was obtained from both cases and their close family members (i.e., parents or siblings). Since all FEP cases included in this study had an illness onset >16 years, only childhood and early adolescence PAS subscales were completed to minimize the possibility that behavioral aspects related to the prodromal phase of the illness are captured [45]. In accordance with previous studies, premorbid period was defined as ending one year before the manifestation of positive psychotic symptoms [46, 47]. For each developmental stage, academic and social domain PAS scores were estimated, with higher score denoting poorer PA. In the PICOS study, the Premorbid Social Adjustment (PSA) scale was administered to assess both academic and social functioning aspects during childhood (5-11 years) and adolescence (12-16 years) developmental periods [48].

#### Premorbid intellectual ability

In the AthensFEP study, premorbid intelligence quotient (IQ) was estimated using the standardized score of the vocabulary subtest of the Greek version of Wechsler Adult Intelligence Scale – Fourth Edition (WAIS-IV) [49, 50], which is tapping the crystallized type of general intellectual ability and therefore it is considered an acceptable neuropsychological proxy measure of premorbid intelligence. Vocabulary subtest performance is not expected to differ depending on the severity of psychopathology [51] and it is largely resistant to neurological and psychological impairment [52]. Premorbid IQ was assessed in PICOS using the Italian version of the National Adult Reading Test (NART), which is commonly used to measure premorbid intelligence in clinical settings [53].

### Genotyping and data processing

DNA extracted from blood samples in the AthensFEP study were genotyped at the Centre for Inherited Disease Research (CIDR) genomics facility at the Johns Hopkins University School of Medicine (Baltimore, USA), using the Infinium Omni2.5Exome BeadChip genotyping array (Illumina Inc., San Diego, USA), which covers >2.4 million genetic markers across the human genome. Whole-genome genotyping of DNA samples in the PICOS study was performed at the Institute of Psychological Medicine and Clinical Neurology at Cardiff University (UK), using custom Illumina Human-CoreExome-24 BeadChip genotyping arrays containing probes for 570.038 genetic markers. In both studies, genotype calling was conducted with the Genome Studio software package and genotype data were further processed for standard quality control (QC) using PLINK v1.09 [54] and custom Perl scripts. Individual samples with low genotype call rates (<95%), genotypic sex inconsistencies, heterozygosity, and cryptic relatedness were excluded from subsequent analyses. In addition, non-autosomal single-nucleotide polymorphisms (SNPs), SNPs with poor genotyping rate (<98%), strand ambiguity (A/T and C/G SNPs) or reference mismatch, low minor allele frequency (MAF < 5%), and Hardy–Weinberg equilibrium deviations (*p* < 10^−6^) were excluded from the final dataset. Following QC filtering procedures, a total of 205 unrelated cases from the AthensFEP and 123 cases from the PICOS study with available clinical and genomic data were successfully genotyped for 1.278.386 and 559.505 autosomal SNPs, respectively. A principal components analysis (PCA) was conducted to control for potential population genetic sub-structure or ancestry related differences and the top four principal components were included as additional covariates in the association analyses.

### Polygenic Risk Scoring

In both study cohorts, polygenic risk scores for circulating 25-hydroxyvitamin D levels (PRS-vitD) were computed based on the summary statistics from the largest to date genome-wide association study (GWAS) on vitamin D concentration [30] (summary data for BMI-adjusted vitamin D levels were analyzed), utilizing the PRSice-2 software [55]. The PRSice software calculates PRSs for each individual applying the clumping and thresholding (C + T) method, which weighs each genetic variant based on the reported effect size for this variant in the corresponding GWAS meta-analysis (i.e. discovery dataset) and computes an aggregate score by summing weighted scores for all analyzed variants in the independently genotyped sample (i.e. target dataset). Different sets of SNPs were filtered in both cohorts by applying increasing *p-*value thresholds (*P*_T_) to the discovery GWAS summary statistics and appropriate linkage disequilibrium (LD)-based SNP clumping (SNPs with *r*^2^ > 0.1 in 250 kb-windows were removed) was performed to ensure that only independent genetic markers are included in the computed PRS. SNPs within the major histocompatibility complex (MHC) LD region on chromosome 6 (hg19; chr6:27-33 Mb) were excluded from PRS computation due to the high polymorphic nature of this genomic region.

### Statistical analysis

Socio-demographic and clinical characteristics were compared between FEP cohorts using Pearson’s chi-squared test for dichotomous variables and independent samples t-test for continuous variables. Linear regression models were applied to assess the relationships between standardized PRS-vitD at each *P*_T_ threshold value, PANSS derived subscale scores and PA domain scores (i.e. academic, social). Regression models were adjusted for appropriate covariates, including gender, age, clinical site and top principal components (PCs) to control for potential ancestry-dependent population stratification. Estimations for the proportion of the explained variance (adjusted-*R*^2^) were calculated by subtracting the variance explained by covariates from the full model incorporating both PRS and covariates as predictors. Sensitivity analyses were also performed by applying multivariable regression models to examine confounding effects related to medication exposure at baseline (i.e. days of antipsychotic treatment at baseline), duration of untreated psychosis (DUP) in weeks, and ICD-10 based consensus diagnoses (F20.0 vs. non-F20.0 diagnosis). Following adjustment for potential confounders, the estimated standardized coefficients (β values) and *R*^2^ values were compared between unadjusted and adjusted regression models. Associations surpassing the nominal statistical significance threshold at *p* < 0.05 (two-tailed) in primary analyses within the AthensFEP were tested for independent validation in the PICOS. To further account for the statistical burden of multiple comparisons in the AthensFEP discovery cohort, nominal *p*-values obtained from association testing were corrected according to the Benjamini-Hochberg false discovery rate (FDR) method (FDR cut-off was set to 5%) [56]. All the analyses were conducted using appropriate functions for linear statistical modeling from the *stats* and *QuantPsyc* packages as implemented in R version 4.1.2 (http://www.r-project.org).

## Results

### Effects of vitamin D PRS on clinical characteristics

Demographic and clinical information from the AthensFEP (total *N* = 205) and PICOS (total *N* = 123) cohorts is summarized in Table 1. Within each FEP cohort, PRS-vitD did not differ between males and females at any of the examined PRS *P*_T_ thresholds (AthensFEP *p* > 0.40; PICOS *p* > 0.50). Moreover, no evidence for an association between PRS-vitD and DUP was observed in both FEP cohorts (AthensFEP *p* > 0.30; PICOS *p* > 0.10). Similarly, there were no PRS-vitD differences between FEP cases that received a schizophrenia diagnosis (AthensFEP 54%, PICOS 24%, compared to the remaining cases with a non-schizophrenia diagnosis (*p* > 0.10 in both cohorts).

**Table 1:** Sample characteristics of the AthensFEP and PICOS first-episode psychosis (FEP) cohorts.

	Discovery cohort AthensFEP	Validation cohort PICOS	Test Statistic (*p*-value)
Participants (total N)	205	123	n/a
Male gender (%)	67	59	*χ*^2^ = 20.44 (*p* < 0.001)
Mean age (±SD)	26.0 (7.6)	30.0 (8.9)	*t* = −4.415 (*p* < 0.001)
Antipsychotic-naïve at baseline (%)	58.4	8.8	*χ*^2^ = 28.90 (*p* < 0.001)
ICD-10 Schizophrenia diagnosis (% F20.0)	54	24	*χ*^2^ = 28.13 (*p* < 0.001)
Duration of untreated psychosis (Mean ± SD, in weeks)	30.1 (54.0)	17.0 (30.7)	*t* = 3.049 (*p* = 0.003)
**PANSS subscale scores, baseline** (Mean ± SD)			
Positive	28.4 (6.9)	21.3 (7.0)	*t* = 8.922 (*p* < 0.001)
Negative	20.2 (8.4)	18.2 (9.8)	*t* = 1.887 (*p* = 0.060)
General	49.5 (13.8)	43.5 (13.2)	*t* = 3.926 (*p* < 0.001)
Total	98.0 (23.5)	82.9 (23.2)	*t* = 5.686 (*p* < 0.001)
**PANSS subscale scores, follow-up**^**§**^ (Mean ± SD)			
Positive	14.6 (5.9)	10.7 (4.7)	*t* = 6.582 (*p* < 0.001)
Negative	14.3 (6.7)	13.1 (7.5)	*t* = 1.451 (*p* = 0.148)
General	30.6 (9.6)	27.1 (10.2)	*t* = 3.101 (*p* = 0.002)
Total	59.5 (19.3)	50.9 (19.7)	*t* = 3.867 (*p* < 0.001)

*ICD-10* International Classification of Diseases 10th Revision, *PANSS* Positive and Negative Syndrome Scale.

^§^Follow-up assessment performed at 4-weeks in the AthensFEP and at 1-year in the PICOS.

### Association between vitamin D PRS and symptom severity

**Fig. 1:**
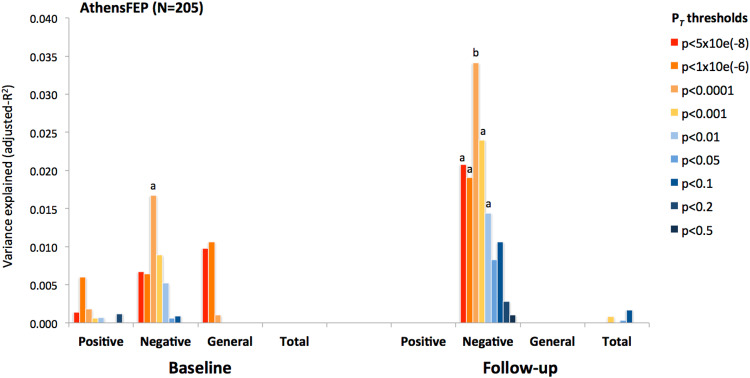
Association between polygenic risk score (PRS) for vitamin D levels and symptom severity in the AthensFEP study. The variance explained (*R*^2^) for PANSS subscale scores by different PRS-vitD *P*_T_ thresholds is shown at baseline and after 4 weeks of antipsychotic treatment (^a^*p* < 0.05, ^b^*p* < 0.001).

**Fig. 2:**
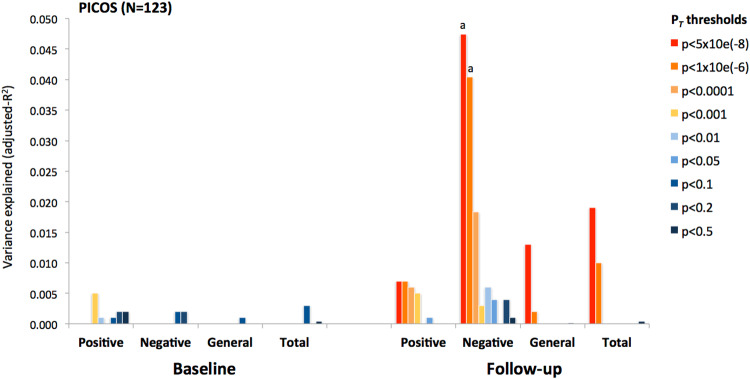
Association between PRS-vitD and symptom severity in the PICOS study. The variance explained (*R*^2^) for PANSS subscale scores by different PRS-vitD *P*_T_ thresholds is shown at baseline and after 1 year of antipsychotic treatment.

We first evaluated the impact of PRS-vitD on distinct symptom dimensions within the AthensFEP at baseline (antipsychotic naïve state) and following 4-weeks of antipsychotic treatment. As shown in Fig. 1, lower PRS-vitD (best PRS *P*_T_ < 1e-04) correlated with more pronounced negative symptoms at baseline and follow-up (baseline: β = −0.15, *p* = 0.03, adjusted-*R*^2^ = 1.8%; follow-up: *β* = −0.20, *p* = 0.003, adjusted-*R*^2^ = 3.4%), however the estimated significance did not surpass the cut-off for multiple comparisons. No significant associations were detected with positive symptoms, general psychopathology, and total PANSS scores at both time points. In order to independently validate the PRS-vitD effect on negative symptoms, we examined the associations between PANSS subscale scores and PRS-vitD in the PICOS cohort (Fig. 2). We observed that lower PRS-vitD predicted more severe negative symptoms at follow-up assessment (follow-up at 1-year), explaining almost 5% of phenotypic variance (PRS-vitD at *P*_T_ < 5e-08 β = −0.24, *p* = 0.008, adjusted *R*^*2*^ = 4.9%; PRS-vitD at the previous optimal threshold *P*_T_ < 1e-04 β = −0.17, *p* = 0.059, adjusted *R*^*2*^ = 2.1%), which corroborates the AthensFEP results.

### Lower vitamin D PRS associated with impaired social motivation

**Fig. 3:**
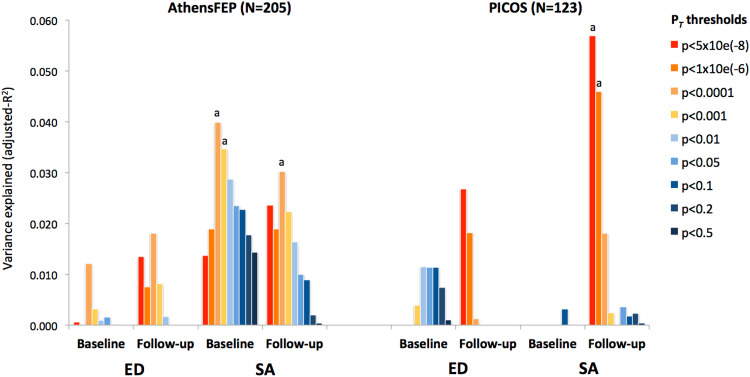
Association between PRS-vitD and PANSS-derived negative symptom sub-domains in the AthensFEP and PICOS studies. The variance explained (*R*^2^) for Expressive Deficits (ED) and Social Amotivation (SA) symptom subdomains by different PRS-vitD *P*_T_ thresholds are shown at baseline and follow-up assessments.

To further elucidate the relationship between reduced PRS-vitD and negative symptomatology, two PANSS negative symptom dimensions, namely expressive deficits (ED) and social amotivation (SA) were examined [42, 57]. In the AthensFEP, a significant association was observed for SA dimension at baseline (PRS-vitD at *P*_T_ < 1e-04: β = −0.24, *p* = 0.0007, FDR-adjusted *p* = 0.024) and a near significant association at follow-up, following adjustment for multiple testing (PRS-vitD at *P*_T_ < 1e-04: β = −0.19, *p* = 0.0046, FDR-adjusted *p* = 0.072). A much weaker nominal association was noted for ED sub-domain (baseline: β = −0.14, *p* = 0.045; follow-up: β = −0.15, *p* = 0.027). In accordance with the above results, lower PRS-vitD predicted increased ED and SA scores at follow-up in the PICOS cohort, indicating a greater impact on SA dimension (PRS-vitD at *P*_T_ < 1e-08: ED β = −0.19, *p* = 0.04; SA β = −0.25, *p* = 0.0037). Associations between PRS-vitD and negative symptom dimensions are summarized in Fig. 3.

### Vitamin D PRS effect on premorbid IQ and functioning domains

As insufficient vitamin D levels have been implicated in aberrant brain developmental processes, we examined the potential impact of PRS-vitD on premorbid intelligence (estimated total IQ) and premorbid adjustment (PA) domain scores. The results from sub-samples of both cohorts with available premorbid IQ scores and PA academic and social domain assessments (AthensFEP *N* = 164; PICOS *N* = 97), did not reveal significant associations with the examined phenotypic outcomes (Table 2).

**Table 2:** Association between PRS-vitD, premorbid IQ and adjustment indices.

	AthensFEP (*N* = 164)		PICOS (*N* = 97)	
	PRS^a^ adj-*R*^2^	*p*-value	PRS^b^ adj-*R*^2^	*p*-value
**Premorbid IQ**^**c**^	0.002	0.587	0.001	0.835
**Childhood PA**				
Academic	0.005	0.338	0.002	0.288
Social	0.005	0.791	0.008	0.602
**Early adolescence PA**				
Academic	0.007	0.117	0.017	0.099
Social	0.002	0.184	0.001	0.804

^a^Denotes PRS-vitD computed at GWAS *P*_T_ < 1E-04, ^b^PRS computed at GWAS *P*_T_ < 5E-08.

^c^Premorbid IQ scores estimated with WAIS vocabulary subtest in AthensFEP and NART in PICOS.

*PA* Premorbid Adjustment, *IQ* Intelligence Quotient.

### Evaluation of antipsychotic treatment and duration of untreated psychosis

In order to ensure that the observed relationship between PRS-vitD and negative symptoms among FEP cases is not confounded by exposure to antipsychotic medication (i.e. induction of secondary treatment-related negative symptoms), we compared severity of negative symptoms in drug-naïve or minimally treated (<10 days), and medicated (>10 days) FEP cases. In the AthensFEP, antipsychotic-naïve cases (58% of total cases) were characterized by more severe negative symptoms at baseline (β = 0.22, *p* = 0.010), compared to minimally medicated cases. The above association was not evident at 4-weeks follow-up (β = 0.07, *p* = 0.442). Similar results were obtained in PICOS, which largely included medicated cases at baseline (mean days of antipsychotic treatment: 22 days), and a much smaller number of antipsychotic-naïve cases (9%). No significant differences in negative symptoms severity were observed at baseline between medicated and minimally treated cases (<10 days 42% of total cases; β = 0.09, *p* = 0.313). With regard to duration of untreated psychosis (DUP), no substantial confounding effect was noted in the results from the primary analyses in both cohorts, when testing follow-up PANSS negative subscale score as the outcome (AthensFEP: PRS-vitD at *P*_T_ < 1e-04: β = −0.20, *p* = 0.0025; PICOS: PRS-vitD at *P*_T_ < 5e-08: β = −0.21, *p* = 0.028).

## Discussion

To the best of our knowledge, this is the first study reporting a relationship between genetic predisposition to reduced vitamin D levels and psychotic symptomatology among individuals diagnosed with psychosis-spectrum disorders. Specifically, the study provides novel and replicable evidence for an association between common variant polygenic risk for lower 25-hydroxyvitamin D concentration and greater severity of negative symptoms in two independent cohorts of individuals with FEP. As PRS-vitD has not previously been implicated to increased risk for schizophrenia, the results of the present study suggest that suboptimal levels of vitamin D often observed in individuals with psychotic disorders [25, 58], do not essentially represent a risk factor for psychotic disorder development, rather common genetic variants associated with lower vitamin D predispose to more severe negative symptoms, in particular avolition and asociality. It is noted that the underlying biological mechanism which might explain the association between PRS-vitD and social motivation cannot be inferred from the results of this study. It could be argued that the PRS-vitD aggregates the effect of multiple genes implicated in biochemical processes that determine vitamin D concentration, as the original GWAS for vitamin D levels has identified several variants located within genes coding for protein products involved in metabolic pathways relevant to vitamin D physiology [30]. Consequently, it may be assumed that the same genetic loci could predispose to the exacerbation of negative symptoms in patients with psychosis. The above notion is also in agreement with recent evidence from genome-wide analyses supporting the common genetic architecture between vitamin D levels and schizophrenia liability [29].

Nevertheless, additional research is needed to delineate the exact mechanism by which reduced PRS-vitD is associated with social motivation in clinical populations and most importantly examine the possibility of confounding effects related to environmental influences and lifestyle factors that might cause fluctuations of vitamin D levels. As abnormal social motivation generally impedes psychosocial performance and persists in patients with schizophrenia [59, 60], the early detection of motivation difficulties preferably during first psychotic episode in vulnerable individuals (i.e. carriers of low PRS-vitD) would considerably ameliorate illness progression and long-term clinical outcomes. In the case that reduced PRS-vitD is associated with less vitamin D as the result of behavioral traits linked to diminished vitamin D production, it is stressed that the current findings suggest that carriers of lower PRS-vitD present with more social motivation deficits owing to behavioral features that hinder outdoor activities, physical exercise, and social relationships. Since PRS-vitD may not directly influence the abundance of circulating vitamin D levels, it is acknowledged that future investigations could provide more insight into the exact biological or environmental underpinnings of the association between PRS-vitD and negative symptomatology in patients with FEP.

Amotivation or apathy represents a core negative symptom in psychotic disorders [61, 62], likely reflecting abnormal emotional management [63] and is often associated with functional disability and poor clinical outcomes [43, 64–67]. The neurobiological correlates of amotivation/apathy implicate reward processing dysfunction and aberrant goal-directed behaviors [68, 69], involving functional disruptions of the ventral striatum brain region and fronto-striatal circuits [70, 71]. More recent conceptualization of negative symptom dimensions links amotivation and lack of pleasure to behavioral disturbances related to positive affect and psychosocial dysfunction, whereas expressive deficits (i.e. blunted affect, alogia) are seemingly more strongly associated with neurocognitive ability [72]. Of note, mounting evidence has implicated inadequacy of serum vitamin D levels to a greater severity of negative and depressive symptoms among individuals with either FEP or schizophrenia [13, 15, 17, 19, 20, 22, 24]. Furthermore, less improvement of negative symptoms over time has been linked to reduced vitamin D levels in drug-naïve patients with FEP [23]. It would be of great interest to pursue clinical research to clarify whether PRS-vitD might be utilized as a predictor of negative symptom severity in the early phases of psychosis, as well as in chronic schizophrenia that is frequently characterized by treatment-resistant, enduring negative symptoms [73–76].

Our findings reveal a genetic relationship between vitamin D levels and social amotivation dimension, implying a greater impact of vitamin D insufficiency on avolition/apathy and asociality symptoms rather than on expressive deficits dimension. This finding is in line with prior evidence indicating an association between low prenatal vitamin D levels and deficient social and emotional development among infants [77]. Moreover, reduced vitamin D levels during pregnancy or early childhood has been considered a risk factor for the development of autism-spectrum disorders (ASD), known for the development of severe social communication difficulties [78]. However, the lack of vitamin D PRS association with premorbid indices considered as promising indicators of compromised neurodevelopment (premorbid IQ, premorbid adjustment), does not support a direct link between polygenic liability for reduced vitamin D concentration and impaired neurodevelopmental trajectories in psychotic disorders. As the genetic contribution of PRS-vitD on neurodevelopmental processes is currently unknown, it is argued that much larger and well phenotyped clinical samples are required to robustly assess the involvement of vitamin D polygenic effects on neurodevelopmental abnormalities that have been associated with pronounced negative symptoms [79].

A number of limitations need to be taken into consideration when interpreting the findings of this study. First, it is important to stress that the observed findings were derived from two FEP cohorts with a relatively small sample size and further confirmation is needed in larger samples of individuals with FEP or psychosis-spectrum disorders in order to robustly implicate decreased PRS-vitD in motivation and social functioning difficulties. Nevertheless, the independent replication of the contribution of PRS-vitD on negative symptom severity in the AthensFEP and PICOS cohorts provides some evidence of reliability to the current findings. Second, a causal role of PRS-vitD on negative symptoms could not be justified in this study, as measurements of vitamin D levels are not available for the individuals examined. Thus, it is not possible to elucidate whether PRS-vitD effect is mediated through the reduction of vitamin D concentration or genetic predisposition for lower vitamin D levels impacts on negative symptoms via pleiotropic effects. Third, this study cannot accurately determine whether lower PRS-vitD exerts an influence on primary enduring or secondary negative psychotic symptoms [75]. It is acknowledged that as the AthensFEP and PICOS cohorts differ in terms of treatment exposure at baseline and follow-up assessment of symptom severity, it is difficult to elucidate whether PRS-vitD influences primary or secondary negative symptoms in each cohort. The fact that a consistent association between low vitamin D genomic burden and social amotivation dimension was observed in both cohorts at follow-up assessments denotes that genetic risk for low vitamin D could predispose to persistent negative symptoms that are typically resistant to antipsychotic medication.

## Conclusions

Motivational impairment and asociality could result in social functioning adversities, low quality of life, and inadequate functional remission in individuals with psychotic disorders [64, 65, 80]. The present study suggests that common genetic variation associated with decreased vitamin D might serve as a potential indicator of motivation and sociability impairment during the early course of psychosis. Future population-based genomic studies within clinically-assessed cohorts of individuals with psychosis could elucidate whether low PRS-vitD designates polygenic vulnerability linked to aberrant metabolic processes or apparently points to indirect influences of non-specific behavioral traits associated with insufficient vitamin D production, such as limited sun exposure, lack of physical activity, and poor nutrition. Pending additional validation from independent studies, the utility of PRS-vitD as a marker of greater risk to develop enduring symptoms of social amotivation and withdrawal might enhance the overall social functioning of individuals with FEP through personalized therapeutic interventions, which will encourage outdoor life experiences, habitual physical activity, and social engagement.

## Data Availability

GWAS summary statistics for circulating 25-hydroxyvitamin D (25OHD) concentration [30], utilized in the present study are publicly available via the following link (https://cnsgenomics.com/content/data). Data involving personal or clinical information are not publicly available. Access to data related to the findings of this study is available on reasonable request from the corresponding author.
